# Experience and challenges from clinical trials with malaria vaccines in Africa

**DOI:** 10.1186/1475-2875-12-86

**Published:** 2013-03-04

**Authors:** Grace Mwangoka, Bernhards Ogutu, Beverly Msambichaka, Tutu Mzee, Nahya Salim, Shubis Kafuruki, Maxmillian Mpina, Seif Shekalaghe, Marcel Tanner, Salim Abdulla

**Affiliations:** 1 Ifakara Health Institute, Ifakara, Tanzania; 2 Malaria Clinical Trials Alliance (MCTA), INDEPTH Network, Accra, Ghana; 3 Swiss Tropical and Public Health Institute, Basel, Switzerland

**Keywords:** Malaria, Vaccines, Clinical trials, Experiences, Challenges, Africa

## Abstract

Malaria vaccines are considered amongst the most important modalities for potential elimination of malaria disease and transmission. Research and development in this field has been an area of intense effort by many groups over the last few decades. Despite this, there is currently no licensed malaria vaccine. Researchers, clinical trialists and vaccine developers have been working on many approached to make malaria vaccine available.

African research institutions have developed and demonstrated a great capacity to undertake clinical trials in accordance to the International Conference on Harmonization-Good Clinical Practice (ICH-GCP) standards in the last decade; particularly in the field of malaria vaccines and anti-malarial drugs. This capacity is a result of networking among African scientists in collaboration with other partners; this has traversed both clinical trials and malaria control programmes as part of the Global Malaria Action Plan (GMAP). GMAP outlined and support global strategies toward the elimination and eradication of malaria in many areas, translating in reduction in public health burden, especially for African children. In the sub-Saharan region the capacity to undertake more clinical trials remains small in comparison to the actual need.

However, sustainability of the already developed capacity is essential and crucial for the evaluation of different interventions and diagnostic tools/strategies for other diseases like TB, HIV, neglected tropical diseases and non-communicable diseases. There is urgent need for innovative mechanisms for the sustainability and expansion of the capacity in clinical trials in sub-Saharan Africa as the catalyst for health improvement and maintained.

## Introduction

Every year approximately 250 million clinical malaria cases are reported [1] with about one million deaths in sub-Saharan Africa, mostly in children under five years of age [2]. If children survive multiple infections due to malaria, such exposure leads to semi-immunity that limits the severity of the disease later in life. However, this immunity wanes in the absence of continued exposure to malaria. Additionally, pregnant women and newborns have reduced immunity and, therefore, are vulnerable to severe complications of malaria infection and disease with poor outcome [3,4].

The currently available tools for the control of malaria are largely, early diagnosis and prompt treatment of clinical episodes with effective anti-malarial drugs. Other tools include the promotion and use of insecticide-treated nets (ITNs) as well as limited use of indoor residual spraying (IRS) in an integrated manner tailored to the socio-ecological setting. An effective malaria vaccine would greatly complement available control measures and accelerate the efforts towards achieving the elimination and eradication goal. The research and development agenda is to develop vaccines that can serve as key components of a future arsenal of tools to eradicate malaria [5]. Current efforts to develop malaria vaccines are primarily directed towards reducing the morbidity and mortality that are associated with malaria and focus on *Plasmodium falciparum*. Malaria Vaccine Roadmap [6] has a strategic goal of developing a vaccine with 80% protective efficacy against *P. falciparum* by 2020.

However, if malaria vaccines are to contribute to programmes for malaria elimination, they will need to have an impact on malaria transmission. The strategies should focus on the development of vaccines that can be used in concert with other malaria control interventions to interrupt malaria transmission and eventually contribute to the eradication of this disease. Moverover, vaccine development efforts need pay attention to Plasmodium species other than *P. falciparum*, especially *Plasmodium vivax*, if malaria eradication is to be achieved [5].

Recent years have witnessed considerable effort from international organizations to support and finance different malaria control strategies aimed at control and elimination of malaria; these efforts include malaria vaccine development. This has led to a robust pipeline of malaria vaccine candidates in the last decade. Several African research institutions took centre stage in the evaluation of these candidates including the front runner, RTS,S. The largest phase III malaria vaccine trial on RTS,S involving 16,000 children is underway in 11 research centers in seven African countries. The initial results of the phase III, RTS,S/AS01 has shown to reduce severe malaria by approximately 50% in older age group of 5 to 17 months and 36.6% in the young children ( 6–12 weeks)[7,8].

This paper discusses the need for more effective tools to control and eliminate malaria, and the challenges, opportunities, and experiences encountered during evaluation of malaria vaccine candidates in Africa. Such information is useful for future product Research and Development (R&D) and to inform stakeholders in planning and implementing future vaccine and drug trials.

### Challenges in selecting suitable malaria vaccine candidates

Identification of malaria vaccine candidates together with the understanding of the pathogen disease mechanism and host immune response interactions has been a major challenge. This is because of the complex life cycle of the parasite. Moreover, *P. falciparum* presents numerous antigens that could feasibly be targets of protective responses. However, such antigens are most often polymorphic, and even exhibit clonal variation through differential multigene expression.

Despite these challenges, efforts have been made to develop malaria vaccine candidates. As of December 2012, WHO updated the rainbow table spread sheet indicating the number of vaccine candidate that are in clinical phase development and those still in preclinical phases [9].This is a great achievement in vaccine development whereby more vaccine have advanced to the clinical phase. However, this may also show inability to predict protection induced by a particular candidate vaccine in early phases hence, substantially increasing the risks of investment by forcing investigators to make “go” decisions based on immune measures tested to decide if the approach is promising in large scale trials [10].

### Challenges in the implementation plan for vaccine trials

#### The correlates of malaria immunity

Vaccines are probably the single most cost-effective public health intervention, past, present and possibly future, to control emerging and re-emerging infectious diseases. A vaccine is given in order to stimulate the development of adaptive immune responses to fight a particular pathogen against which the vaccine has been developed. Regardless of the success in the development of malaria vaccines, there is still a lack of understanding of individual immunity against malaria. Since the work of Koch on Java Island at the end of the 19th Century, which showed that adults who survive malaria infection acquire a highly effective immunity, the mechanisms involved and how they operate remains partly unknown, although the antibody that blocks the invasion of merozoites into erythrocytes appears to play a crucial role [11].

Some work has been done to determine which protective antigens or epitopes can be used in the construction of recombinant, subunit or synthetic malaria vaccines [12-14]. Surrogate markers of antibody efficacy currently rely on in vitro assays that are laborious and difficult to reproduce, and it remains unclear if such in vitro assays are predictive of functional immunity in humans due to the lack of suitable animal model permissive for *P. falciparum*[15]. The work leading to antibody-dependent cellular inhibition (ADCI) is an important cue in the case presented in this commentary on the need of more human evidence based hypothesis. This approach led to studies to correlate clinical protection in an endemic population with the immune responses to malaria vaccine candidates in development and gave insights into the relative proportion of cytophilic antibodies to non-cytophilic antibodies (the C:NC ratio) as being the most important surrogate marker of protection to date[16-18].

Currently, there are no clear correlates of immunity against pre-erythrocytic and blood-stage parasites. Immuno-assays can be validated only once a vaccine demonstrates efficacy in a clinical trial. Once an immune correlate for protection is identified, it can be used for decision making in clinical development. Immunoepidemiological studies have demonstrated that immunity against blood stage *Plasmodium falciparum* is associated with the acquisition of anti-parasite antibodies of the cytophilic subclasses [19], and in particular IgG3 [18,20-26].

Recently, it has been shown that, there is an association between the frequency of RTS,S/AS01E induced (circumsporozoite protein) CSP-specific CD4+ T cells and protection from clinical malaria, most strongly seen for IFNc-IL2-TNF + CD4+ T cells. Furthermore, there were significant interactions between CSP-specific TNF + CD4+ T cell responses and anti-CSP antibodies induced by RTS,S/AS01E vaccination. This interaction suggests that the protection afforded by the combination of CD4+ T cells and anti-CSP antibodies is greater than would be predicted by their sum [27].

RTS,S vaccine candidate induces high concentrations and frequencies of antibodies and CD4+ T cells, respectively, specific for circumsporozoite protein (CSP) [28,29]. Anti-CSP antibodies correlate with protection against infection in malaria naïve adult challenge studies [28] and field studies in young children [30], against clinical malaria in trials with young children in Kenya/Tanzania [31] and in Gabon/Ghana/Tanzania [32], but anti-CSP antibodies did not correlate with protection against clinical malaria in a trial with older children in Mozambique [33]. Anti-CSP antibodies could protect by a variety of mechanisms including complement activation, antibody dependent cellular cytotoxicity, sporozoite neutralization, and/or FccR mediated phagocytosis [34].

Immunologic analyses indicate that high titre anti-CS IgG are most strongly associated with RTS,S-mediated protection, with an important additive component from CS-specific Th1 cells. One recent study highlighted a correlation between CS-specific TNFa(+) CD4 (+) T cells and reduced morbidity, which requires confirmation in other studies [31]. The first results from the Phase 3 trial were published and were in line with expectations from the Phase 2 trials [7,35], expect for the young age group which provided modest protection against malaria [8]. A likely explanation for the lower vaccine efficacy among infants is an age-dependent differential immune response to the vaccine. This concept is supported by the lower anti-circumsporozoite antibody titers observed in infants as compared with titers in older children reported previously [7].

Infants may have mounted a lower immune response than older children owing to coadministration of RTS,S/AS01 with routine EPI vaccines, an inhibitory effect of maternally derived anti-circumsporozoite antibodies, an absence of priming with hepatitis B vaccine or with *P. falciparum* infection, or the infant’s immature immune system. Coadministration of RTS,S/AS01 with the pentavalent vaccine and the oral poliovirus vaccine might have resulted in immune interference and contributed to the lower anti-circumsporozoite antibody titers in the younger infants [8].

There are many lessons to be learned from the RTS,S trials including the major contribution of sporozoite challenge trials, the importance of adjuvant, dose and schedule optimization [36].

In a Phase 2a experimental sporozoite challenge trial in malaria non-immune Caucasian volunteers, vaccine related partial but modest protection against sporozoite challenge was observed in terms of a delay in time to parasitaemia [37], although no sterile protection was observed. A recently completed Phase 1b vaccine trial in semi-immune Tanzanian adults and children confirmed the safety and immunogenicity of the platform. In addition, an exploratory analysis showed a reduced incidence of clinical episodes of malaria. As interesting as it is, this requires confirmation in field efficacy studies [38].

Other vaccines based on irradiated sporozoites or genetically modified attenuated sporozoites have provided protection in challenge models [39]. Such whole organism attenuated vaccines may provide effective protection against malaria and significantly reduce parasite transmission. Over 1,000 bites by the irradiated mosquitoes per volunteer were required for consistent protection against challenge. Importantly, there have been no breakthrough *P. falciparum* infections in volunteers immunized by sporozoites irradiated with > 120 Gray units. Protection against challenge lasted for at least 42 weeks (10 months) after the last immunization. Furthermore, studies have shown that volunteers who are exposed to infected non-irradiated mosquitoes while taking chloroquine develop durable protection [40,41]. However, considerable technological challenges in terms of manufacturing, formulation, and delivery of such attenuated sporozoite vaccines need to be overcome [5].

As for vaccines that target the sexual stage of the parasite, they do not aim to prevent illness or infection in the vaccinated individual, but to prevent or decrease transmission of the parasite to new hosts. This ‘transmission-blocking’ vaccine can be seen as a true altruistic vaccine [12]. Previous clinical trials of sexual stage vaccines that have been discontinued involve ookinete antigens Pfs25 from *P. falciparum* and Pvs25 from *P. vivax*. In both studies and in pre-clinical work by the same group there is a consistent correlation between titre of anti-Pfs25 antibody and membrane-feeding assay (MFA) activity [42].

Nonetheless, in the absence of surrogate measures of protection conferred by these vaccines, probably the best way to assess the effectiveness of a developed vaccine is by conducting clinical trials in natural conditions. For a vaccine to be effective, it must elicit the appropriate immune responses that will protect the individual from future infections or disease.

The understanding of the immune correlates will provide the missing piece of puzzle to improve the performance of RTS,S and to fully optimize other vaccine candidates. The ongoing phase III RTS,S vaccine trial is a unique opportunity, with African scientists playing a central role in assessing not only efficacy of the vaccine, but also its mode of action and correlate(s) of protection. Exploration of factors that might affect vaccine efficacy, including the effect of maternal antibodies, the role of immune interference by EPI vaccines, the effect of the RTS,S/AS01 booster, and status with respect to previous exposure to *P. falciparum* parasites, will provide crucial information for the further development of this vaccine and for other malaria vaccines under development [9].

Tackling such questions leads to additional research capacity (resources both human and infrastructure) development in the field of basic human immunology and systems biology. It will further foster the GCP-ICH and good clinical laboratory practice (GCLP) approaches through standardization of approaches, methods and procedures.

#### Clinical trial end points

Suitable choice of the primary end points in the controlled trials is critical for each phase of clinical development of the vaccine. For example, the ongoing phase III RTS,S malaria vaccine trial has well-defined and harmonized end points. This allows comparability of the performance of the same intervention in different locations, age groups and over time at the same location [43].

The investigation of relationship between parasite density and likelihood of clinical disease can help to develop a model of specificity and sensitivity for end point definition. Standardization of one method that might have been developed during previous studies is desirable for accuracy, precision and key to comparisons across trials and intra-trial (different sites). Based on this, laboratory procedures for malaria parasite quantification in RTS,S trial has been harmonized across sites to ensure both accuracy and to allow for comparability throughout the trial [44]. This includes, for instance, similar standard operation procedures (SOPs) for slide reading and interpretation, a key determinant of study end point in a malaria intervention trial [45].

Selection of the appropriate clinical trial efficacy end points (e g, risk of developing a disease or severe disease) is of crucial importance and calls for standardized protocols of malaria case definition for current and future trials or studies adapted to the different levels of care where the protocols will be applied (e g, dispensary *versus* hospital). Similarly, phase IV trials will also require full standardization of other key end points, such as health system factors or cost-effectiveness and cost-benefit assessments.

#### Monitoring of malaria transmission intensity

The malaria control strategies implemented in many endemic areas have resulted in a decrease of malaria transmission in many parts of Africa and elsewhere [46]. Despite these efforts, vulnerable children, under five years of age, are still at risk of dying from malaria disease. This calls for the continued search for a malaria vaccine, which could complement the existing integrated control strategies. Being able to measure accurately malaria transmission is a key factor for any control programme, as well as measuring the impact of new control tools as identified by the MalERA-process [47]. Concurrent assessment of malaria transmission intensity during malaria vaccine trails is important in the interpretation of efficacy results by providing accurate information on the endemicity and seasonality of malaria transmission. Accurate data on transmission will also help in the design of phase IV studies, deployment of the vaccine and subsequently effective surveillance-response systems in order to be able to evaluate and understand the relationship between transmission intensity and health outcomes in a given area. Unfortunately there is still a lack of appropriate methodological approaches.

Data generated through microsimulation [48,49] proposed an algorithm estimation of human infection rates from the entomological inoculation rate (EIR). Infant mortality rates decrease markedly when the EIR is reduced, probably largely because of prevention of indirect mortality. It was also observed that reduction of exposure to malaria during infancy is not reflected in increased mortality at older ages, a concern of many who think that good childhood protection programmes may predispose to later susceptibility [48]. Modeling is only one of the avenues to be pursued for quick and better capturing transmission with changing levels of endemicity; new approaches involving biomarkers and particularly serology needs to be explored [47]. However, modeling cannot replace real-time monitoring of transmission during studies.

### Challenges and opportunities during implementation

#### Clinical care as benefit to the community

In Africa, clinical trials are usually conducted in communities with little or limited access to health care facilities and even if health facilities are present they often suffer from limited resources, such as personnel, infrastructure and medical supplies. Although it is well established that any trial, irrespective of trial outcome, will contribute to a general improvement in the health infrastructure and the care provided, sponsors are not readily willing to cover the additional cost to support/improve the health care of the community where trials are conducted despite this being a real need. Investigators should make a collaborative effort to develop a policy of supporting health care for the community where trials are being conducted and this policy should be communicated to sponsors during the planning stage.

Increasing the effectiveness and efficiency of health care services is important everywhere but particularly in developing countries with limited resources [50]. Clinical audits are an example of a useful tool in improving clinical care that should be adopted by African investigators. The provision of quality care is a key factor in the success of clinical trials with regard to community acceptance, compliance and adherence to protocol. This ensures that the trial execution changes positively the health-seeking behavior of the community concerned. Therefore any clinical trial that is well conducted and well grounded in the study community will impart long lasting health benefits.

#### Follow-up and adherence to the study protocol

Adherence to the study protocol in clinical trials is the essential prerequisite for both the investigators and the study participants. Failure of safety evaluation by the investigators or loss of follow-up among study participants obviously creates major setbacks in research findings. Loss of follow-up can be due to many reasons (death, illness, worsened health, and refusal, withdrawal, side effects and general dissatisfaction by participants with regard to trial conduct) [51]**.** A well-planned and conducted informed consent at individual and community level can substantially reduce withdrawals and losses to follow up [52]. Participants may not comprehend all the information provided in the informed consent due to the poor consenting process or literacy status of the participant or misinformation about research in the community. Engaging the community and participants is cited as an element for a successful retention of participants in a study**.** The establishment of community advisory boards (CAB) to enhance community understanding of study and procedures of research lead to mutual trust and a sense of collective ownership [53-57] and have become an integral part of studies with long follow-up periods. Sponsors and investigators should, therefore, make efforts to enhance two-way communication with communities to discuss issues that may be of concern to both parties.

Modern technology has helped to overcome poor infrastructure in African trial settings. Participants can now be reached through mobile phones to enable research teams to ask parents the health status of their children, remind them of pending visits and schedule emergency care. A simple questionnaire can be used to evaluate health status via mobile phones, which are reachable throughout the year, even during rainy seasons, and reduce the problem of loss to follow up due to poor roads. Proper planning and risk analysis by the investigators and sponsors before implementation is the key to successful evaluation of the vaccine within African communities. Geo-referencing has become a key tool in knowing the location potential of study participants, distance to health facilities and other physical landmarks that are of interest to specific research. This technology, linked to continuous demographic surveillance, provides an optimal framework for planning and executing large studies, but comes with additional costs for which sponsors have to be prepared.

The sharing of new information regarding the vaccine under investigation is necessary and another way of providing incentives for participants and an avenue for sharing both negative and positive research results that are important for future trials, it also keeps participants loyal to the investigator and *vice versa*.

#### Harmonization of the ethical review process

Given the diversity of ethical review committees (ERC) in Africa, there is a high likelihood of diverse decisions if the same study protocols are submitted to these committees, particularly in a growing number of multicentre studies. However, the decision-making process of the individual countries and committees must be respected. The need to harmonize ethical review processes is urgent. Harmonization could first focus on procedural aspects of the ethical review process and subsequently address substantive aspects of ethical review, which could be more challenging than the former. A participatory approach to include all interested stakeholders is critical for harmonization to be acceptable to African ERCs and also to be effective in terms of improving the quality and timing of the review process without compromising on science. During the process, clear roles and responsibilities of national ERCs in relation to institutional ERCs in countries where both national and institutional committees exist, should be stipulated and agreed by all involved. The role of the committees should be complementary rather than duplicative. Such clarity would go a long way towards minimizing potential antagonism between national and institutional ERCs of the same country [58].

Capacity building for the committee members is of equal importance, as it is crucial that ethical committees reviewing protocols are adequately knowledgeable about all established national and international guidelines. AMANET, was among Africa’s research NGOs, made an effort to promote health research in Africa that meets international scientific and ethical standards. AMANET took a holistic approach to address infrastructural, human and financial resource needs of African research institutions [59]. For example, the Ifakara Health Institute (IHI) Ethical Review Committee in Tanzania secured a sub-grant from AMANET which was used to start a two-way community engagement programme aimed at conveying information about health research ethics to the communities in one of its centres involved in clinical trials, Bagamoyo [60]. The programme enhanced the ERC’s and the researchers’ understanding of community concerns and perceptions on health research [61,62]. This approach by the ERC of engaging communities to complement efforts that may be made by researchers could go a long way to encouraging participatory approach in health research so as to ensure that participants and their communities are treated as stakeholders who should be kept informed about research conducted on them.

With the current interest and investment in conducting clinical trials to test different investigational products in African countries, there is a need to consider establishing pre-IND meetings, where researchers invite members of different ethical committees and regulatory authorities to discuss the possibility of conducting a potential study in order to speed up the process before investing on such trial.

The harmonization of procedures is inevitable to be able to adequately support multicountry studies. This will be more evident as product development takes root in Africa with local scientists leading the development of products that have to undergo different stages across different countries because none of the countries is endowed with the capacity for all the processes to support product development.

#### Capacity building

Today, different vaccine candidates have advanced in clinical trials (phase II to III). These vaccine candidates are being evaluated in a variety of transmission and epidemiology settings so as to demonstrate efficacy across the board. A great push for evaluation of new malaria prevention tools in malaria-endemic settings created the drive to strengthen clinical research capacity in Africa, both in human and physical infrastructure.

In Bagamoyo, Tanzania, for example, the IHI-site has been able to conduct the malaria vaccine trial through the use of a new, well-equipped research laboratory, a renovated paediatric ward and enhanced telecommunications, thanks to support from the INDEPTH-Malaria Clinical Trials Alliance (MCTA) and MVI. The Swiss Tropical and Public Health Institute has been the site partner through the process from planning the trial facility to date. The site also managed to establish a quality assurance team that provides internal quality monitoring to ensure compliance to the protocol and site-standard operation procedures in line with GCP requirements.

MCTA has been working in partnership with Malaria Vaccine Initiative (MVI), and the Medicines for Malaria Venture (MMV) to support clinical trials site in Africa in various aspects. Bagamoyo clinical trial site is one among other African sites that received support from MCTA for personnel training, acquisition of equipment and infrastructure upgrades to ensure the successful execution of clinical trials. MCTA has enabled African clinical research sites to establish and equip laboratories with state-of-the-art equipment, improve communication systems, database management, financial and managerial systems, including human resources management. Patient care and management has also been improved through the use of digital X-rays which enable the clinical team to improve diagnostics accuracy and service, not only for trial participants but also for the community at large [62]. Providing scientists and medical staff with access to quality facilities and equipment/technologies helps to retain highly trained personnel, this in turn contributes significantly to the improvement of quality of health care services at national level.

Moreover, to fast tract capacity development, clinical trial scientists or trialists need to have a recognized, viable career path or professional development in the African region, as in Europe, USA and Asia. Conceptualizing a clinical question, developing an appropriate protocol and ultimately conducting the trial to answer that question is a research discipline that requires training, experience and critical thinking. Availability of a critical mass of skilled clinical trial coordinators, clinical trial auditors, laboratory technicians, data managers, statisticians, clinical research nurses, and clinical trial physicians and investigators will make the African research centers take a lead in the design and execution of their own programmes [63].

#### Sustainability of the clinical trial centers

Presently, few clinical trial centers exist in Africa that can conduct ICH-GCP clinical trials. To ensure optimal evaluation of the growing number of malaria vaccine and drug candidates, not forgetting other diseases that are in the pipeline, the available capacity must be strengthened and expanded. This will ensure sustainability of the centers while making sure their relevance in scientific research is maintained in the dynamic clinical landscape. Knowledge in biomedical ethics is also needed to ensure standards are applied throughout any clinical development process of products using varied technological innovations that become available, such as genetic engineering and nanotechnology.

The conduct of clinical trials is complex - not only in terms of products being tested, but also in balancing scientific and ethical obligations to study populations. The capacity to ethically review study protocols and provide ethical oversight of clinical trials is a core component of responsible research systems. Each country and major institution involved in the conduct of clinical trials should have adequate capacity to conduct such ethics review. EDCTP through MARC project (Mapping of ethics review and trial regulatory capacity in sub-Sahara Africa) supported strengthening of the existing ethical review capacity of health research responsible to conduct EDCTP funded clinical trials throughout Africa [64].

Career development and job security are required to attract and retain African scientists to ensure there is a critical mass of competent, skilled personnel available to support these centers in the long term.

Finally, there is need for investment in capacity development to perform long-term follow up of trial subjects, including phase IV studies, to provide the information on the rare events that may occur following the use of new products and those currently being developed. This in turn suggests that such trial sites be linked to a health and demographic surveillance system such those found on the INDEPTH network, to ensure optimal longer term follow up of populations involved in studies. This is going to be critical for safe and effective monitoring at all levels. For example, the concept of evaluating RTS,S in phase IV should be planned in advance and budgeted for as part of the risk management plan at registration with regulatory authorities. Phase IV for RTS,S will be a breakthrough and will bring a lot of knowledge that might have been missed in previous and ongoing malaria vaccine evaluations. This will also expand the scope of the already established INDEPTH Network Effectiveness and Safety Surveillance (INESS) embedded within the existing HDSS platforms in Mozambique, Tanzania, Ghana and Burkina Faso.

Besides the elements of human, physical and social (community involvement) capacity building, sustainability of a trial centre will only be assured once the centre develops a full trial portfolio that goes beyond a single disease aspect and single disease research programme, e g, a mid- to long-term portfolio that includes basic science, drug and vaccine trials, for example, malaria, TB and HIV, neglected diseases, non-communicable diseases for different population groups (infants, children, adults). This will allow continuous participation of African scientists in finding new approaches and solutions to African and global health problems.

#### Outlook

There are several malaria vaccine candidates and the majority of these vaccines are based on recombinant proteins and over one-half consist of a single antigen [9]. With limited financial resources available to evaluate these candidates, scientists have to prioritize or develop more define selection criteria to select vaccine candidates to trial. Also, there is a requirement for different clinical trial phases to be planned in advance. For instance, facilities to conduct challenge studies, preclinical and phase I **–** where the experimental infection of malaria-naive volunteers by the bite of *Plasmodium falciparum*-infected mosquitoes has been a preferred means to test the protective effect of malaria vaccine and drug candidates in malaria-naive volunteers, is not the same as for phase IV. State-of-the-art facilities for clinical care, laboratories, expertise in terms of personnel need a proper budgeting and preparation prior to implementation.

A systematic, evidence-based approach for prioritizing vaccine candidates would expedite the development of a specific candidate. The criteria for selection of a promising vaccine will provide a clear pathway and promote greater confidence among scientists and funders that investments are focused on the best candidates. This approach could facilitate collaboration between African scientists and pharmaceutical companies in the formulation and development of vaccine candidates. Exchange of expertise will allow African investigators to be involved in protocol development and study design, eventually building confidence and the basis to participate in phase I, which is usually conducted in developed countries. GSK Biologicals, Rixensart and Novartis has been supporting fellowship programmes which offer opportunities within the malaria young African investigators/clinicians to get on job training in various aspect of clinical trials and vaccine development. These research training programmes are aimed at developing sustainable partnerships with select scientific institutions in emerging countries to further this endeavor [65]. The establishment of phase I and challenge units in malaria-endemic countries will expedite product development and allow screening of products early in the target population allowing for optimization of the candidates before the conduct of field trials. This will also ensure these centers participate in the full breadth of product development.

Developments over the past decade, as well as the number of successfully completed malaria drug and vaccine trials, clearly shows the active participation of African scientists in African research centers in the evaluation of new tools against diseases of poverty. This should provide the framework for moving phase I and challenge studies to the region and ultimately make Africa a key player in developing innovative solutions to alleviate Africa’s disease burden and spur development.

Additionally, researchers conducting clinical trials in developing countries have ethical obligations beyond those falling on researchers working in the developed world, such as ensuring access to standards of care and ensuring the rights of study participants are held as the oversight systems are not well developed. No single profession, team, or country has a monopoly on wisdom. Establishing partnerships will assure procedural fairness and promote the ethical conduct of clinical trials in a world characterized by grave inequities.

## Competing interests

The authors declare that they have no competing interests.

## Authors’ contributions

SA, GM and BM have made contributions to conception and development of the manuscript. GM led the writing of the manuscript, coordinating all the reviewers’ comments. BO, MT, SS, TM, NS, MM and SK have critically revised the manuscript. All authors read and approved the final manuscript.
